# Comparative field study of silver nanoparticles and garlic oil nanoemulsion for nematode control and yield enhancement in eggplant

**DOI:** 10.1038/s41598-025-06697-0

**Published:** 2025-06-20

**Authors:** Hamida A. Osman, Hoda H. Ameen, Mahmoud E. Abd El-Aziz, Usama S. Elkelany

**Affiliations:** 1 Plant Pathology Department, National Research Centre, El-Behooth St, Dokki, Giza, 12622 Egypt; 2 Polymers and Pigments Department, National Research Centre, El-Behooth St, Dokki, Giza, 12622 Egypt

**Keywords:** Eggplant, Bio silver nanoparticles, *Bacillus cereus* Nem 212, Garlic-oil nano emulsion, *Meloidogyne incognita*, Biological techniques, Biotechnology

## Abstract

The root knot nematodes *Meloidogyne incognita* are the most serious threats affecting eggplant, *Solanum melongena* L. Chemical nematicides are commonly used against plant-parasitic nematodes, but due to their negative impact on health and the environment, safe and environmentally friendly alternatives have become increasingly important. Biological control using rhizospheric bacteria metabolites, and plant substances have, emerged as important alternatives to the use of agrochemicals. Recently, nanotechnology has revolutionized nematode management and its effectiveness in biological control. The aim of this study is to evaluate the efficiency of *Bacillus cereus* Nem 212 supernatant and garlic essential oil in their regular and nanoscale forms against *M. incognita* infecting eggplants CV. Baladi and investigate their impact on plant growth parameters under field conditions. All nano formulations produced higher nematicidal activities compared to their respective original extracts. Garlic oil nano emulsion and garlic oil emulsion were more effective as natural nematicides on *M. incognita*, and in enhancing eggplant growth parameters, followed by bio- silver nanoparticles synthesized by *B. cereus* Nem 212 filtrate. These formulations are promising and environmentally friendly alternatives for controlling root knot nematode and have the potential to reduce reliance on hazardous agrochemicals. In addition, these techniques increase vegetative plant growth and yield production.

**Supplementary Information:**

The online version contains supplementary material available at 10.1038/s41598-025-06697-0.

## Introduction

Eggplant *Solanum melongena L*., is one of the most popular vegetable in Egypt, 90% of the eggplant yield is produced by five countries: China, India, Iran, Egypt and Turkey^1^. Eggplant is a warm-season vegetable but can grow in Egypt at different seasons^2^. In each100 gm of eggplant there is1.4 gm protein, 18 mg calcium, and 24 kcal of food energy^3^. Eggplant production is affected by the infestation of several pests including plant-parasitic nematodes (PPN), which are among the most serious threats to vegetable crops, especially the root knot nematodes RKN *Meloidogyne* spp. *Meloidogyne incognita* is one of the most common *Meloidogyne* species worldwide, affecting crops in warmer climates^4^. This pest is a serious problem because it can attack many different plants and interact with harmful organisms into the soil to cause complex diseases. Additionally, it has the ability to suppress the plant immunity or decrease the plant resistance to other pathogens, or weaken plants, making them susceptible to mild pathogens. This pathogen causes significant economic losses up to 80%^5^ by altering the plant vascular system, draining essential nutrients from the host and developing the root knot galls. The damage caused by these nematodes can result in stunted growth, root deformation, reducing plant resistance to other biotic and abiotic stresses related to other problems such as drought, nutrient deficiency as well as economic losses for the growers^6^ Chemical nematicides are commonly used against plant-parasitic nematodes. However, due to their negative impact on health and the environment the use of these nematicides has been either heavily regulated or completely banned from agriculture^7^. Nowadays, due to the growing demand for safe and healthy food products, environmental friendly alternatives to manage plant parasitic populations have become increasingly important. Biological methods of control have emerged as an important environmental friendly alternative to the use of agrochemicals and can provide satisfactory control of root-knot nematodes in vegetables and other crops. Among numerous organisms that have shown antagonism against RKN, without negative effects on users, consumers and the environment are Plant-Growth Promoting Rhizobacteria (PGPR). They reduce nematode population when colonize the plant host rhizosphere and promote plant growth^8^. The most studied rhizobacteria are *Bacillus* spp. The culture filtrate of the rhizospheric bacterium *Bacillus cereus* Nem 212 have been reported to inhibit RKN reproduction, egg hatch, juvenile survival, and promoting plant growth by producing different enzymes and toxins^9^. In addition to the microorganisms, plants are promising sources of substances to solve the problems resulting from the extensive use of the synthesized nematicides as some of their metabolites can be used directly against nematodes. Among these metabolites essential oils are reported to be active against nematodes. Garlic essential oil and its volatile components have repeatedly been studied. The antimicrobial properties of garlic extracts have already been investigated and were attributed to the organosulfur compound allicin^10^.

Recently, nanotechnology has revolutionized nematode management and its effectiveness in biological control. These materials, which have a size smaller than 100 nanometers, provide better protection against pollution. Some of the benefits that have been attributed to biosynthesized nanoparticles (NPs) include their ability to enhance plant growth, reduce root galling, and cause mortality of motile stages of nematodes, thus by constituting an effective form of biological control^11^. Silver nanoparicles (Ag-NPs) have demonstrated efficiency against many plant pathogens, including nematodes while having less negative impacts on human, animals and environment^12,13^. The nematicidal activities of nanoparticles (NPs) against root-knot nematodes have been mentioned in some studies. Cromwell et al.^14^ found that silver nanoparticles (30–150 µg/mL) caused inactive *M. incognita* j2. Taha and Abo-Shady^15^ reported that 1500 ppm of (Ag-NPs) achieved 96.5% mortality of larvae after 72 h of treatments. In vitro study Nazir et al.^16^ found that the silver nanoparticles (Ag-NPs) possess nematicidal activity against RKN and can act as an alternative to high-risk synthetic nematicides, or inconsistent biological control agents without causing any phytotoxicity. Tariq et al.^17^ reported that biologically synthesized AgNPs provide significant nano-enabled features for broad-spectrum agricultural applications, specifically in plant pathogen inhibition, because they exert antibacterial, antifungal, and antinematic potential. The antifungal and antinematicidal activities of chemically and biologically synthesized silver nanoparticles (chemo-Ag-NPs and bio-Ag-NPs), as well as an aqueous extract of *Moringa oleifera* leaves, were evaluated in vitro against *Macrophomina phaseolina* and root knot caused by *M. incognita*, the results indicated that bio-Ag-NPs exhibited greater efficacy than chemo-Ag-NPs under greenhouse conditions, (bio-Ag-NPs) significantly reduced the incidence of damping-off and charcoal rot caused by *M. phaseolina*, as well as decreased the number of *M. incognita* juveniles in the soil, along with a reduction in gall formation and egg mass production, compared to untreated control plants^18^. Mohammad et al.^19^ evaluated the nematicidal effects of *Artemisia annua* and *Lepidium sativum* extracts, in both original and nano forms, against *M. incognita* in tomato under greenhouse conditions. The nano-formulations, characterized by smooth, spherical particles (50–100 nm), exhibited greater nematicidal activity than the original extracts under laboratory conditions. Nanoparticle formulations of extractives of *Urtica urens* recorded up to 11-folds increase in the nematicidal activity against root-knot nematode *M. incognita* when compared to their corresponding raw extractives^20^. Hamed et al.^21^ reported that the biosynthesized Ag/AgCl-NPs exhibited significant reduction of eggs hatchability at low concentrations (10–20% v/v) as well significant increase in larval mortality over the chemical nematicide at concentrations (50–500 ul/L). El-Habashy^22^ found that nanoparticles form of *lantana camara*, and *conyzadio scoridis* consistently showed higher effects than normal extracts forms. Hamed et al.^23^ provided a promising technique for green production of (Ag-NPs) extract of the heterocytous cyanobacterium *Nostoc* sp. PCC7524. The study indicated that the nematicidal activity of the bacteria on egg hatching, and larval mortality of the root –knot nematode *Meloidogyne javanica* in vitro and under green-house conditions, which suggest their use in biological control of *M. javanica*. The study also highlighted the positive role of cyanobacterium- based (Ag-NPs) in improving some plant growth parameters cultivated in nematode-infected soil. Therefore, cyanobacterium- based (Ag-NPs) could be suggested as multifunctional nano-nematicide to avoid the harmful effects of the chemical nematicide, and could be used as part of an integrated program for controlling some plant diseases. However, employing (Ag-NPs) in the soil is controversial due to their impact on biosystem, e.g., changing the soil bacterial diversity^24^ and protected wheat plants from heat stress and improved plant growth and biomass^25^. Pandey et al.^26^ and Hojjat^27^reports that (Ag-NPs) enhanced seed germination, seedling vigor and increased shoot length, root length, number of leaves and other plant growth criteria of different crops. Dietz and Herth^28^ found a decrease in carbohydrates and protein content, while AL-Huqail et al.^29^ found an increased in accumulation of proline in *L. termis* L. seedlings.

The aim of this study is to evaluate the efficiency of the rhizospheric bacteria *Bacillus cereus* Nem 212 and garlic essential oil in their regular and nanoscale forms against the root knot nematode *Meloidogyne incognita* infecting eggplant, CV. Baladi and investigate their impact on plant growth parameters under field. (Table 1).

**Table 1:** Treatments designed for controlling root-knot nematode *Meloidogyne incognita* infecting eggplant CV. Baladi under field conditions.

Treatments number	Frequency of application	Treatment full name	Application rate		Treatments abbreviation
		A-Normal formulations			
T1 control		Untreated infected control (negative control			
T2- a b	1 2	Silver nitrate solution at planting Silver nitrate solution at planting and after one month	10 ml/plant 10 ml/plant		AgNO_3_
T3- a b	1 2	Endophytic bacteria *Bacillus cereus* Nem 212 Cell free culture filtrate at planting Endophytic bacteria *Bacillus cereus* Nem 212 Cell free culture filtrate at planting and after one month	10 ml/plant 10 ml/plant		*B.cereus* cell free filtrate
T4- a b	1 2	Garlic essential oil emulsion at planting Garlic essential oil emulsion at planting and after one month	10 ml/plant 10 ml/plant		garlic oil emulsion
B- Nanoparticles formulations (Dose, and time of application as normal formulation)					
T 5- a b	1 2	Bio-silver nanoparticles synthesized by* Bacillus cereus *Nem 212 filtrate at planting Bio-silver nanoparticles synthesized by *Bacillus cereus* Nem 212 filtrate at planting and after one month		10 ml/plant 10 ml/plant	bio-silver nanoparticles Bio - Ag-NPs
T6- a b	1 2	Garlic oil nanoemulsion at planting Garlic oil nanoemulsion at planting and after one month		10 ml/plant 10 ml/plant	garlic oil nanoemulsion GaO- nanoemultion GaO-NPs

Frequency of application: 1- at planting time 2- at planting time and after one month.

## Results

### Effects of application frequency of endophytic bacteria and garlic oil emulsion in their regular and nano form against nematode *Meloidogyne incognita* reproductive parameters

**Fig. 1:**
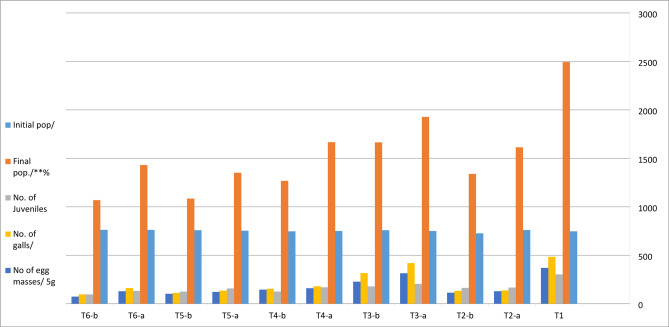
Effects of endophytic bacteria extracts in both normal and bio-silver nanoparticles, garlic oil emulsion and nanoemulsion on eggplant CV. Baladi infested with *Meloidogyne incognita* under field conditions.

Regarding the effects of frequent applications of the bio agents at planting time, and after one month on nematode reproductive parameters, the recorded data indicates that the percentage reduction of the nematode reproductive parameters increased with increasing frequency of application in the treatments i.e. percent reduction is correlated with the frequency of application, as shown in (Table 2; Fig. 1).

Under field conditions the obtained data in (Table 2; Fig. 1) indicates that all tested treatments in both normal and nano- forms at concentration of 10 ml/plant resulted in significant variable (*p* ≤ 0.05) decreases in the nematode reproduction on eggplants compared to untreated control.

At harvest, the treatment T6- b (garlic oil nanoemulsion) provided the highest percentage reduction of 57.2% in the final nematode juveniles in the soil compared to the control. Percentage reductions of 68.2%, 80.2%, 80.2% and 85.1% were recorded in juveniles/ 5 g. roots, number of galls, and egg masses/5 g roots, and total number of eggs/5 g. roots respectively compared to the control.

Followed by the treatment T 5-b (bio-Ag-NPs) that produced the second highest percentage reductions compared to the control. Percentage reductions of 56.4%, 58.6%, 77.1%, 72.3%, and 78.5% were reported in the nematode juveniles in soil, juveniles/ 5 g. roots, number of galls and egg masses/5 g roots and total number of eggs/5 g. roots, respectively compared to the control (Table 2; Fig. 1).

However, the application of T3-a (*B. cereus* Nem 212 filtrate) showed the lowest percentage reductions of 22.7%, 32.1%, 15.5%, 14.9% and 22.5% in the nematode juveniles in soil, juveniles/ 5 g. roots, number of galls and egg masses/5 g roots and total number of eggs/5 g. roots, respectively, compared to control.

Moreover, the treatment T4-a (garlic oil emulsion) exhibited percentage reductions of 33.1%, 44.0%, 63.0%, 56.6%, and 63.1% in the nematode juveniles in soil, juveniles per 5 g. roots, number galls and egg masses/5 g.roots and total number of eggs/5 g. roots, respectively compared to control.

Finally, the application of T2 -a, b (AgNo_3_ solution) resulted in percentage reductions by71.9% and 72.5% in the number of galls/5 g. roots at planting time and at planting and after one month respectively than the untreated control. The percent reduction in total number of eggs/5 g. roots were found to be 71.5 and 74.0% at the same time intervals respectively compared to the control.

**Table 2:** Effects of endophytic bacteria extracts in both normal and bio-silver nanoparticles, Garlic oil emulsion and nanoemulsion on eggplant CV. Baladi infested with *Meloidogyne incognita* under field conditions.

Treatments	*Freq. of app.	Initial pop/ 250 g soil	Final pop./**% 250 g soil Red		No. of % Red. Juveniles / 5 g root		No. of % Red. galls/ 5 g roots		No of egg %Red. masses/ 5 g roots		Total eggs/ %Red 5 g roots	
T1		747a	2493 a	–	302 a	–	484 a	–	369 a	–	212,913 a	–
T2-a	1	760 a	1613 c	26.3	167bc	44.7	136 de	71.9	128 cd	65.3	60672bc	71.5
b	2	726 a	1339 d	46.3	163bc	46.0	133 de	72.5	113 cd	69.3	54240bc	74.0
T3-a	1	750 a	1927 b	22.7	205 b	32.1	419 b	15.5	314 a	14.9	164,850 b	22.5
b	2	758 a	1664 c	33.2	178bc	41.0	316 c	34.7	227 b	38.4	111684bc	47.5
T4-a	1	750 a	1667 c	33.1	169bc	44.0	179 d	63.0	160 c	56.6	48,560 bc	63.1
b	2	747 a	1267de	41.2	125 cd	58.6	154 de	68.2	145 cd	60.7	66,120 cd	68.9
T5-a	1	753 a	1351 d	45.8	157bc	48.0	134 de	72.3	122 cd	66.9	58,316 cd	72.6
b	2	758 a	1085 e	56.4	125 cd	58.6	111ef	77.1	102 cd	72.3	45,696 cd	78.5
T6-a	1	761 a	1431 cd	42.5	132 cd	56.3	161 de	66.7	128 cd	65.3	40320bc	81.0
b	2	762 a	1068 e	57.2	96 d	68.2	96 f	80.2	73 d	80.2	31,536 d	85.1

Each value represents mean of five replicates. Mean followed by the same letter (s) within a column are not significantly (*P* ≥ 0.05) different according Duncan’s multiple range test. *Freq. of app.: Frequency of application: 1- at planting 2- at planting and afterone month. ** % Red: % reduction.

1-T1: Control. T2-a: Silver nitrate at planting. T2- b: Silver nitrate at planting and after one month. T3-a : Endophytic bacteria *Bacillus cereus* Nem 212 cell free culture filtrate at planting, T3-b: Endophytic bacteria *B. cereus* Nem 212 cell free culture filtrate at planting and after one month. T4-a: Garlic oil emulsion at planting. T4-b: Garlic oil emulsion at planting and after one month. T5-a: bio- silver nanoparticles synthesized by *B.cereus* Nem 212 at planting, T5-b: bio-silver nanoparticles synthesized by *B. cereus* Nem 212 at planting and after one month. T6-a: Garlic oil nanoemulsion at planting. T6-b: Garlic oil nanoemulsion at planting and after one month.

### Effects of endophytic bacteria, bio silver nanoparticles, garlic oil emulsion and garlic oil nanoemulsion on eggplant growth parameters

**Fig. 2:**
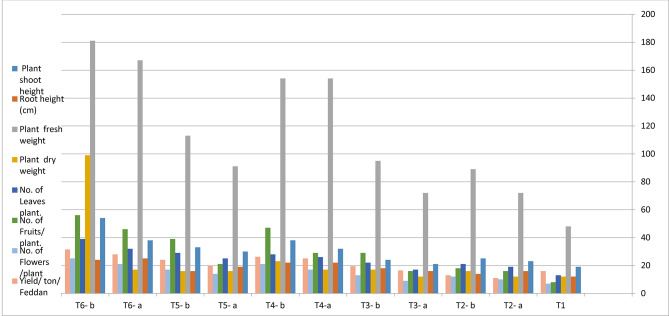
Effects of endophytic bacteria extracts in both normal and bio -silver nanoparticles, garlic oil emulsion and nanoemulsion on eggplant growth parameters CV. Baladi infested with *Meloidogyne incognita* under field conditions.

As for the effects of endophytic bacteria *B. cereus* Nem 212 and garlic oil in their normal and nano forms at concentration 10 ml/plant, on eggplant growth parameters, the recorded data (Table 3; Fig. 2) indicates that all tested treatments significantly improved eggplant growth parameters with variable degrees compared to the control infected untreated plants. The only exception was the application of AgNo_3_ solution (T2 -a, b) decreased fruit weight parameter by -27.0% and − 13.3% compared to the control.

**Table 3:** Effects of endophytic bacteria extracts in both normal and bio -silver nanoparticles, Garlic oil emulsion and nanoemulsion on eggplant growth parameters CV. Baaladi infested with *Meloidogyne incognita* under field conditions.

*Tre.	**Freq of app.	Plant shoot height (cm)	***% Inc.	Root height (cm)	***% Inc.	Plant fresh weight (g)	***% Inc.	Plant ***% dry Inc weight (g)		No. of *** % leaves Inc. /plant		No. of *** % fruits/ Inc. plant		Fruits **** % weight/ change. plant (g)		No. of ***% flowers Inc / plant		Yield/ ton/ Feddan	*** % Inc
T1		19 d	–	12 e	–	48 e	–	12 b	–	13 a	–	8 d	–	750 cd	–	7 e	–	16 cd	–
T2- a b	1 2	23 cd 25 cd	21.0 31.5	16 cd 14e	33.3 16.0	72 de 89 cd	50.0 85.4	12 b 16 b	– 33.3	19 cd 21 cd	46.1 16.5	16 cd 18 cd	100.0 125.0	547e 650 de	−27.0 −13.3	10 cd 12 cd	42.8 71.4	11.0 e 13.0 de	−31.3 −18.8
T3- a b	1 2	21 cd 24 cd	10.5 26.3	16 de 18 cd	33.3 50.0	72 de 95 cd	50.0 47.9	12 b 17 b	33.3 41.6	17 ae 22 cd	23.5 69.2	16 cd 29 bc	100.0 175.0	813 cd 900 c	8.4 20.0	9.0 de 13 bc	28.5 85.7	16.5 cd 19.5 c	3.1 22.0
T4-a b	1 2	32 bc 38 b	68.4 100	22 ab 22 ab	83.3 83.3	154 b 154 b	220.8 220.8	17 b 23 b	41.6 91.6	26 bc 28 bc	100.0 115.3	29 bc 47 a	262.5 487.5	1216 b 1247 b	62.1 66.3	17 b 21 a	142.8 200.0	25.0 b 26.3 b	56.3 64.4
T5- a b	1 2	30 bc 33 bc	57.9 73.7	19 bc 16 cd	58.3 33.3	91 cd 113 c	89.5 125.4	16 b 16 b	33.0 33.3	25 bc 29 bc	92.1 123.0	21 bc 39 bc	162.5 387.0	910 c 950 c	21.3 27.0	14 bc 17 b	100.0 142.8	20.0 cd 24.3 de	25.0 51.8
T6- a b	1 2	38 b 54 a	100.0 184.2	25 a 24 ab	108.3 100.0	167 ab 181 a	247.9 277.0	17 b 99 a	41.6 141.6	32 ab 39 a	146.2 200.0	46 a 56 a	475.0 600.0	1400ab 1757 a	86.6 107.6	21 a 25 a	200.0 257.0	28.0 ab 31.5 a	75.0 96.8

Each value represents mean of five replicates. Mean followed by the same letter(s) within a column are not significantly (p ≥ o. o5) different according Duncan’ s multiple range test *Tre.: Treatments ** Freq. of app. Frequency of applications:1- at planting 2- at planting and after one month. *** % Inc= % increase ****% change: % change of control1-T1: Control. T2-a: Silver nitrate at planting. T2- b: Silver nitrate at planting and after one month. T3-a: Endophytic bacteria *Bacillus cereus* Nem 212 cell free culture filtrate at planting, T3-b: Endophytic bacteria *B. cereus* Nem 212 cell free culture filtrate at planting and after one month. T4-a: Garlic oil emulsion at planting. T4-b: Garlic oil emulsion at planting and after one month. T5-a: bio- silver nanoparticles synthesized by *B.cereus* Nem 212 at planting, T5-b: bio-silver nanoparticles synthesized by *B. cereus* Nem 212 at planting and after one month, T6-a: Garlic oil nanoemulsion at planting. T6-b: Garlic oil nanoemulsion at planting and after one month.

Treated soil with garlic oil nanoemultion (T6-b) resulted in the highest increase of 100%, 200%, 600%, 107.6% 257.0% in the root length, number of leaves, number of fruits, weight of fruits and number of flowers respectively, compared to the control.

The (bio-Ag-NPs) (T5-a, b) increased the length of the plant shoot by 57.9% and 73.7% respectively, while the *B. cereus* Nem 212 filtrate (T3- a, b) increased the same parameter by 10.5% and 26.3% respectively compared to the control.

In addition, eggplants infected with *M. incognita* and treated with (bio-Ag-NPs) (T5-a, b) increased weight of the fruits by 21.3% and 27.0% respectively compared to the control. Meanwhile, the *B.cereus* Nem212 filtrate (T3-a, b) showed increasing in fruits weight by 8.4% and 20.0% respectively compared to the control.

Moreover, the same trend was reported for eggplants infected with *M.incognita* and treated with garlic oil in its nano formulation. The garlic oil nanoemulsion treatment (T6-a, b) increased weight of the fruits by 86.6% and 107.6% respectively although garlic oil solution T4- a, b) resulted in percentage increases of 62.1% and 66.3% in the fruits weight respectively compared to the control.

Upon treating soil with either garlic oil nano formulation (T6-b) or (bio-Ag-NPs) (T5- b) results show that they both have significantly improved the number of leaves by 200% and 123% respectively. Moreover, they both increased the number of fruits by 600% and 387.0% respectively and increased weight of fruits by 107.6% and 27.0% respectively. In addition, both emulsions enhanced the number of the flowers per plant by 257% and142.8% respectively compared to the control plant.

### Effects of different treatments on eggplant yield

All treatments in both normal and nano-forms at concentrations of 10 ml/plant showed a significant increase in eggplant yield compared to the untreated control except application of AgNo_3_ decreased eggplant yield compared to control. The application of AgNo_3_ solution (T2- a, b) decreased eggplant yield by – 31.3% and – 18.8% respectively compared to control (Table 3; Fig. 2).

Treated soil with garlic oil nanoemulsion (T6- a, b) provided the highest percentage increase 96.8% and 75.0% in yield respectively compared to control, followed by the application of garlic oil emulsion (T4- a, b) the percentage increase in yield was 56.3% and 64.4% respectively compared to control. Treated soil with *B. cereus* Nem 212 filtrate (T3-a) exhibited the lowest percentage increase of 3.1%, while the application of (bio-Ag-NPs) (T5-b) resulted in 51.8% increase in eggplant yield (Table 3; Fig. 2).

Moreover, the present study emphasized that all nano formulations produced higher nematecidal activities compared to their respective original extracts. (Fig. 6).

### Characterization of bio- Ag- NPs and gaonanoemulsion

**Fig. 3:**
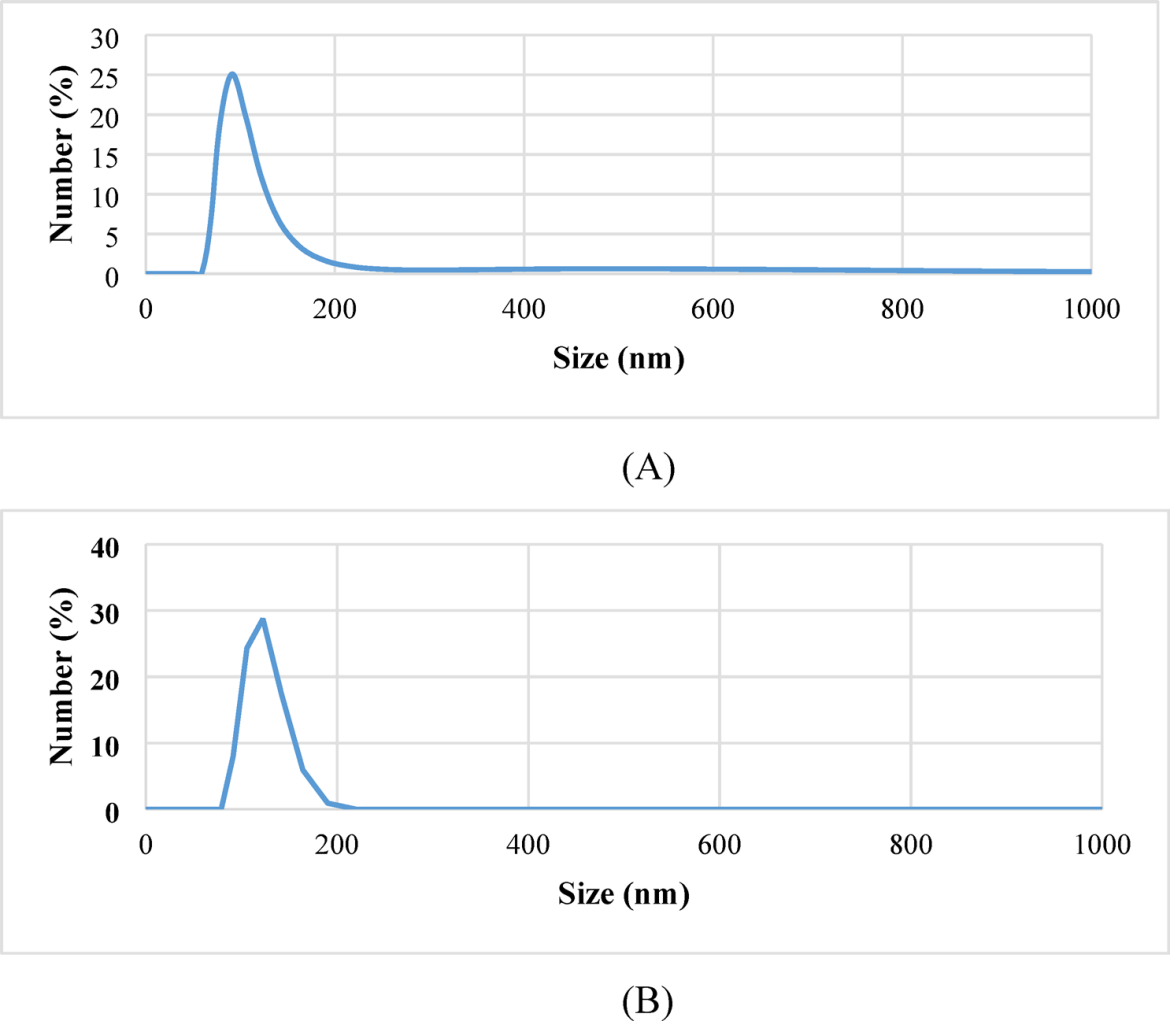
(**A**) Particle size distribution of bio- Ag-NPs. (**B**) Particle size distribution of GaOnanoemulsions.

Figure 3A,B illustrate the particle size distribution of (bio-Ag-NPs) and GaOnanoemulsions, respectively. It can be obtained that the average particle diameter observed was 105 ± 20 nm and 122 ± 27 nm for (bio-Ag-NPs) and GaOnanoemulsion respectively.

**Fig. 4:**
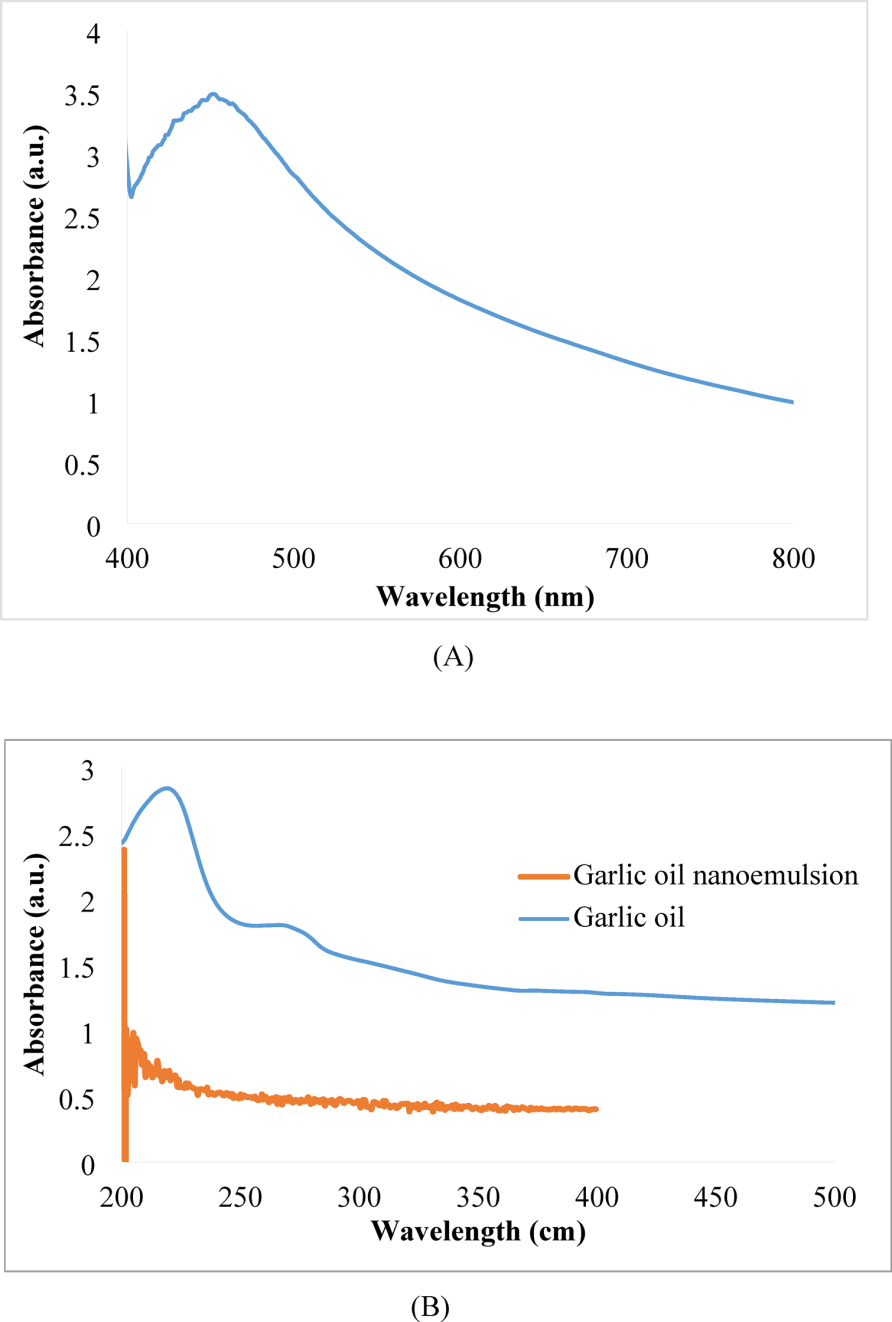
(**A**) UV-Vis spectrum of bio- Ag-NPs. (**B**) UV-Vis spectrum of Garlic oil solution and Garlic oil nanoemulsion.

The synthesized nanoparticles’ UV-visible spectra were captured to identify the surface plasmon resonance (SPR) band. The position, width, and shape of the SPR band can be used to provide a qualitative sense of the size distribution and morphology of the nanoparticles. For example, a broadband signal shows a broad variation of particle sizes, while a single narrow SPR band reveals the presence of spherical monodisperse particles. A large peak at 453 nm is a typical band for (bio- Ag-NPs) as shown in Fig. 4A. This broad SPR peak indicates the presence of irregularly shaped nanoparticles in the solution^30,31^. Figure 4B represent the UV-Vis spectrum of Garlic Oil solution and Garlic Oil nanoemulsion. The garlic oil solution showed a UV-Vis spectrum at 230 nm and no interference of wavelengths of nanoemulsion and garlic oil solution^32^.

**Fig. 5:**
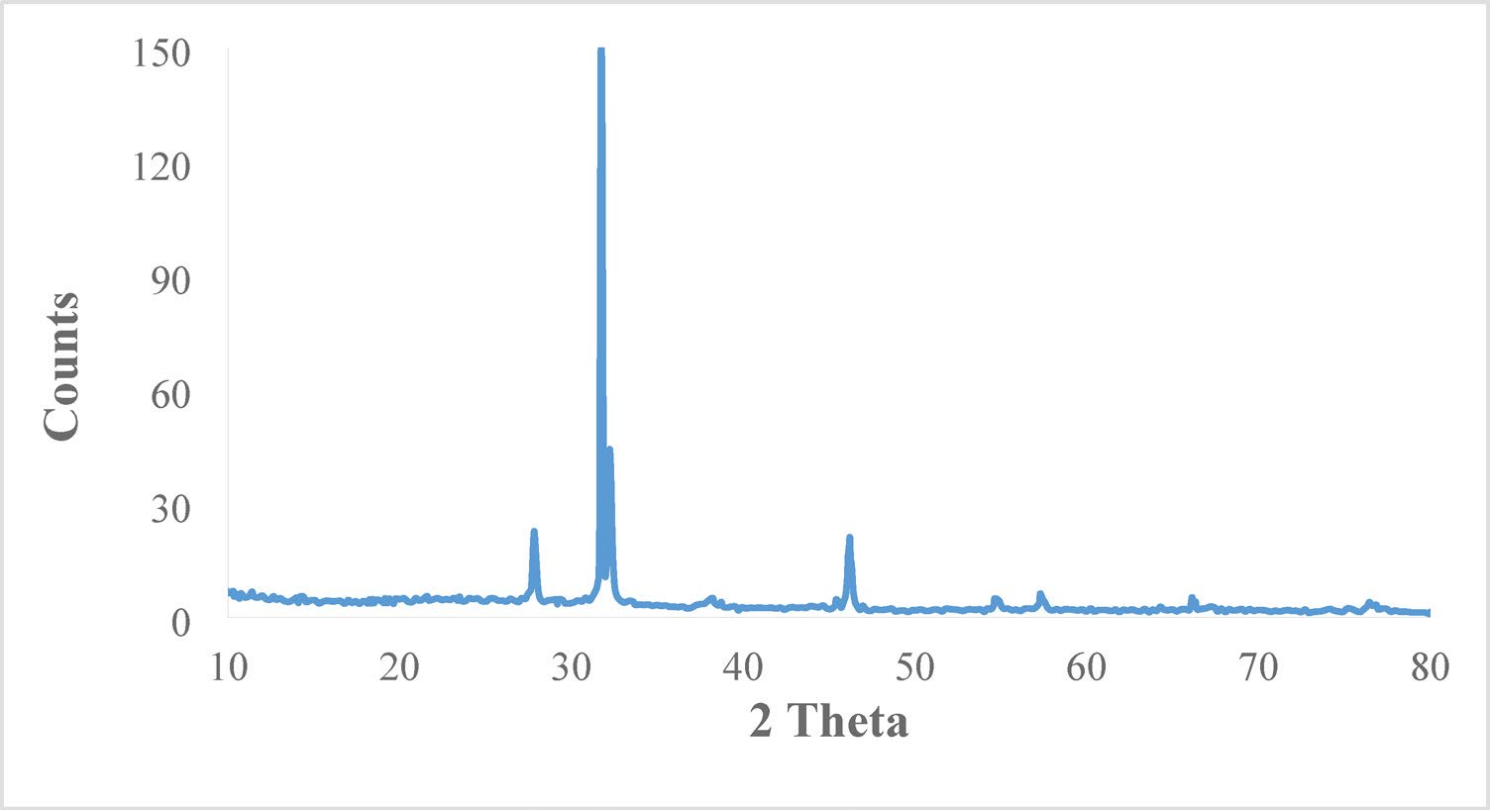
XRD pattern of bio-Ag-NPs.

The XRD pattern of the as-prepared (bio- Ag- NPs) is shown in Fig. 5. It can be shown from Fig. 5 that the peaks of the as-prepared (bio- Ag- NPs) have emerged at 2θ ≈ 38.1°, 46.1°, 67.3°, and 76.4° which assigns to (111), (200), (220), and (311) planes, respectively^33^. This diffraction pattern agrees with the reference (Ag-NPs) (JCPDS file no. 84–0713 and 04-0783). The other peaks in the XRD spectrum indicate the presence of a mixture of silver and silver oxide nanoparticles. Korkmaz and Karadağ^34^ illustrated that Ag_2_O_3_ and Ag_2_O NPs as well as pure (Ag-NPs) were prepared together via a microwave-assisted green synthesis method^35^.

## Discussion

In recent years, the potential of nanotechnology in plant disease management has become the front and center of the biological nematode control literature^36^.

The present investigation has provided evidence that silver nanoparticles synthesized by *Bacillus cereus* Nem 212 cell free culture filtrate resulted in variable significant reductions in the nematode *M. incognita* reproductive parameters, improved the growth parameters and production of eggplant fruits under field conditions. A similar observation was reported^14,23,37^. Nevertheless, AgNo_3_ showed a comparable inhibitory effect against *M. incognita* under field conditions. It also had the lowest effect on improving eggplant growth parameters compared to control infected untreated plants. Jung et al.^38^ attributed the toxic effect of silver ion on *Staphylocoocus aureus* and *Escherichia coli* to membrane dysfunction of the bacterial cell which possibly causes death. The increase in percent reduction in the nematode reproductive parameters was correlated with the frequency of application. Similar patterns of percent reduction of nematode reproductive parameters were observed by frequent application of all the treatments with the bio-agents used in this study either in its normal or their nano forms. These results are in accordance with^8,39^ who revealed that *Bacillus* sp. in its normal form used more than once, or in combination, were more effective than those used once in reducing *M. incognita* reproductive parameters. Application of bio silver nanoparticles enhanced the activity up to 4 times against the number of galls and egg masses of *M. incognita* respectively. Abbassy et al.^40^ showed that increased potency of (Ag-NPs) compared to the reference extractives, and the reference nematicide, which highlights the presence of certain secondry metabolites that cause variable significant reductions in the nematode reproductive parameters. *Bacillus* spp. is one of strains which shows PGPR activity^41^. Rhizospheric microorganisms e.g. plant growth promoting rhizobacteria (PGPR) maintain a close environment around the root of plants that improve plant growth through various mechanisms like N_2_ fixation, solubilization of mineral phosphates, and other essential elements^42^. They play a key role in natural ecosystems and influence plant productivity and inhibition of plant pathogen Osman et al.^43^ and their ability to improve plant nutrient up take, produce phytohormones and siderophores, induce systemic resistance^44^. Xiao et al.^45^ reported that a cell- free filtrate of *B. cereus* decreased *M. javanica* second stage juveniles suggesting that extracellular nematicidal substances were present in the filtrate. Similarly, Tian^9,46,47^suggested that it to be due to extracellular nematicidal substances and by producing different enzymes and toxins.

The utilization of nanoparticles has proven to be effective in the management of several plant parasitic nematodes. Wiesman and Chapagain^20,48^observed that nanoparticlesof up to 100 nm improved properties and activities compared to the original material. This study and based on the present data shows that the average particle diameter obtained was 105 ± 20 nm and 122 ± 27 nm for (bio-Ag NPs) and GaO nanoemulsion, respectively. This result confirms and is in agreement with the data shown by^20,48^. According to Ardakani^33^ the silver and titanium oxide nanoparticles exhibited toxicity towards *M. incognita* in varying concentrations in both in vitro and in vivo. Heflish et al.^35^ stated that the efficiency of (bio- Ag- NPs) using Wilkesiano aqueous leaf extract reduced the nematode activity, mortality, egg hatching, and movement of larvae and could be recommended to manage the plant parasitic nematode *M. incognita* as it is simple, stable, cost- effective, and keeps the environment safe. Soliman et al.^49^ assed the power of polysaccharides and polyphenol extracts as well as their nano- forms from marine algae (*Laurenica papillosa* and *Dilophys fasicola*) for the control of *M. incognita*. They indicated that the tested treatments effectively decreased galls and egg masses of tomato roots compared to control in the field. *D. fasicola* extract and its nano- form showed promising nematocidal activity compared to *L. papillosa* extract. Hassan et al.^37^ observed degradation in cell wall of *M. incognita* juveniles when they were exposed directly to Ag-NPs in water. Toxicity of Ag-NPs is not species – specific, therefore it can be applied to control other plant parasitic nematodes, and plant pathogenic fungi^21^. Its mode of action is associated with disrupting and malfunctioning of several cellular mechanisms, such as membrane permeability, ATP synthesis, and physiological response to oxidative stress in both eukaryotic^49^ and prokaryotic cells^50^.

The inhibitory effect of the bio formed Ag- NPs was attributed to their physical structure (e.g., size, shape, and homogeneity) which, probably played a key role in the cell wall penetration of the nematode eggs, followed by cell dysfunction^51^. Ag- NPs induced oxidative stress and up regulation of sod-3 and daf-12 genes which caused the failure of reproduction in *Caenorhabditis elegans* worms^52^. Silver nanoparticles are nano material being applied as active ingredients of control and their toxicity is due to induction of oxidative stress in the cells of targeted nematodes^53^. Ag-NPs were also found to be effective in improving plant growth. There are reports that (Ag-NPs) enhanced seed germination and increased shoot length, number of leaves, and other plant growth criteria of different crops^26,27,52^. The accumulation and uptake of nanoparticles are dependent on the exposure concentration^27^. Yasmeen et al.^53^ concluded that seeds treatment and incubation time affect the seedling growth.

Based on the data obtained from this investigation, (bio-Ag-NPs) seem to be a good tool for efficient biocontrol of *M. incognita* infecting eggplant, and enhanced plant growth. These results are in harmony with those reported by^12,25^. The bio stimulant effect of either *B. cereus* or (bio-Ag-NPs) on eggplant growth parameters is possibly due to micronutrients and organic compounds such as auxins, giberellins^54^.

The antimicrobial properties of garlic extract have been investigated in the early 19th century and were attributed to the organosulfur compounds found in essential oils such as ajoene, dithiines and diallyl sulphide (DADS) diallyl trisulphide (DATS) and allyl methyl trisulphide that are responsible for its nematocidal activity^55^. The mode of action and the potential cellular targets of diallyl polysulfides are attributed to the different reactive sulfur species. Nematicidal activity of different garlic compositions have been reported against *Meloidogyne* spp. showing comparable efficacy to synthetic nematicides^56^. Different garlic-based pesticides are approved, registered and sold to control nematodes and other pests with different levels of success. Since *Meloidogyne* spp. are endoparasites, a translatable compound that could affect the nematode inside the root plants is desirable. This compound could be found in the essential oils, since it contains several antimicrobial ingredients that work through various mode of action. Nanotechnology is a tool to modify nano–scale material characteristics, to improve the properties of essential oils^57^.

According to the data, the garlic oil nanoemulsion resulted in the highest percentage reduction of 57.2%, 80.2% and 85.1% in the final nematode juveniles in the soil, number galls/5 g roots and total number of eggs/5 g.roots respectively, as compared to control infected untreated ones. Positive effects on eggplant growth parameters were also recorded. Results show significant percentage increase of 200%, 600%, 107.6% and 257% in number of leaves, number and weight of fruits and number of flowers respectively, compared to control. These results are in harmony with Hammad and Hasanin^58^they examined the efficiency of spearmint and thyme essential oils emulsions and nanoemulsions solutions as an alternative to chemical control for suppressing *M. javanica* and *Fusarium oxysporum* on Coleus plant *Coleus forskoblii* in vitro and greenhouse conditions. The results indicated great reductions in the final population of *M. javanica* in the soil. Thyme and spearmint essential oils nano emulsions and emulsions recorded effect on final population (Pf) about 1193.6 and 1465.6, respectively, compared to Fenamiphose (328.4). A similar pattern was discovered in a greenhouse with a positive effect on increased shoot dry weight for coleus plants, where thyme and spearmint oil nanoemulsions at (5000ppm) achieved 3.3 and 3.9 g./plants for root –knot nematode infected plants, respectively, compared to 2.7 and 4.4 g/plants for *F. oxysporum* infected plants.

Essential oils as nanoemulsions or natural nematicides, on the other hand, have a variety of efficiency mechanisms^59^. Mendes et al.^60^suggested, the nano emulsion’santipeptide activity was boosted while cytotoxicity was lowered. Nanoemulsion containing mint essential oil extract and chitosan has nematicidal activity against root knot nematodes with low cytotoxicity in a human cell line^61^. Essential oil emulsion and nanoemulsion components may adversely affect the nematodes’ nervous system, another possibility is that the essential oils disrupt the cell membrane of the nematode, and change its permeability^61^. In soil infested with root knot nematodes, a combination of chitin and benzaldehyde increased tomato plant growth^61^. In addition, sabinene, myrcene and trans- caryophellene concentrations in thyme essential oil nano emulsions, which are a group of terpenoid compounds, sabinene plays a role in nematicidal activity^62^. The gains in plant growth metrics might be attributable to biochemical changes in the stem base tissues, or they could be due to their effectiveness in partially preventing disease infection and development.

Generally, the bacteria *Bacillus cereus* Nem 212 filtrate, and garlic oil emulsion significantly improved the health status of eggplants against nematode infection. Furthermore, these treatments reduced the nematode reproductive parameters (number of galls and egg masses) in eggplant roots. Similar results were recorded in potato plants treated with compost enriched with bacterial cell filtrate of either *B. cereus* Nem 212 or *B. cereus* Nem 213 strains against *M.incognita* infecting potato plants^9^. Garlic essential oil against *M. incognita* infecting tomato plants^55^ and garlic extract formulation against the grapevine nematode *Xiphinema index*^10^. It has been mentioned by^22^ that nanoemulsion proved to be a successful treatment for root- knot nematodes in tomato, with no harmful effects on plants and other non-parasitic nematodes. In addition, emulsion stability improves considerably when the size of the oil particles is lowered^63^. Because essential oils (which have low water solubility) are larger than nano emulsion particles, they cannot easily interact with the cell membranes. However, nanoemulsion particles can deliver essential oils to the surface of the nematode cell membranes, which could be related to the ability of smaller particles to kill or hinder the nematode at any stage of its life cycle^64^.

Our results suggest the efficiency of garlic oil nanoemulsion and bio –silver nanoparticles in root knot nematode control. They provide new trends that safely and successfully prevent or reduce *Meloidogyne* species. Moreover, the present results show that the nano formulation was more effective on *M. incognita* reproductive parameters compared to their original extract. This action was attributed to nanoparticles that may have an inhibiting effect due to their physical proprieties, which was essential for the nematode′s ability to penetrate the cell wall (e.g., body form, size)^65^. Also, this characteristic feature might be due to the high negative- negative repulsion force between the nanoparticles which supports the long–term stability, good colloidal nature, and high dispersity of the silver^66^.

## Conclusions

Overall, the findings of this study demonstrate that garlic oil nanoemulsion and garlic oil emulsion were the most effective natural nematicides against *Meloidogyne incognita*, while also promoting enhanced growth parameters in eggplant. These were followed in efficacy by biosynthesized silver nanoparticles produced using *Bacillus cereus* Nem 212 filtrate. Furthermore, the study highlights that all nano-formulations exhibited superior nematicidal activity compared to their corresponding original extracts.

From the previous results and discussion, it can be concluded, that nanotechnology and the uses of plant extracts, and endophytic bacteria are promising and environmentally friendly methods for controlling root knot nematode that can overcome and replace hazardous chemical nematicides. In addition, these techniques increase vegetative plant growth and yield production.

The utilization of such techniques in root–knot nematode control can provide a safe and effective nematode management program. However, further studies will be required to provide more insights into how this can effectively be utilized as a suitable alternative to chemical nematicides by optimizing formulations, assessing environmental impacts, evaluating efficacy across different crops or nematode species.

## Materials and methods

### Source of seedlings

Seedlings of eggplant (*Solanum melongena* L.) CV. Baladi were obtained from the Department of Horticulture Research Centre, Agriculture Research Centre Giza, Egypt.

### Isolation of bacterial isolate

The bacterial isolate used in this study was isolated from tomato and eggplant farms from Kafr Hakim, village, Giza, Governorate, Egypt, and was identified based on the morpho - taxonomic and molecularly characterized based on the partial 16 S rDNA sequencing analysis by the authors in a previous study^9^. The bacterial isolate was deposited in the GenBank data base as *Bacillus cereus* Nem 212 under accession number OK284601. The bacterial strain was stored on slant of LB broth medium^67^and maintained in Nematode Lab, Plant Pathology Department, National Research Centre, Egypt. A conical flask (250 ml) containing 100 ml of LB broth medium was inoculated and incubated at 28–30 ℃ with shaking at 150 rpm for 48 h. prior to application. After the incubation period, bacterial cultures were centrifuged at 5000 rpm for 15 min. and the supernatant solution was passed through a 0.22 μm in diameter nitrocellulose filter to prepare sterile culture filtrates for further studies.

### In vitro synthesis of silver nanoparticles by *Bacillus cereus* Nem 212 OK284601

Silver nitrate and Tween 20 were obtained from Sigma Aldrich. Garlic oil was bought from the National Research Centre.

### Preparation of silver nitrate solution (AgNO_3_)

Silver nitrate (1 g) was dissolved in 1 L of water to be used in nematode treatments.

### Biosynthesized of silver nanoparticles (bio-Ag-NPs)

**Fig. 6:**
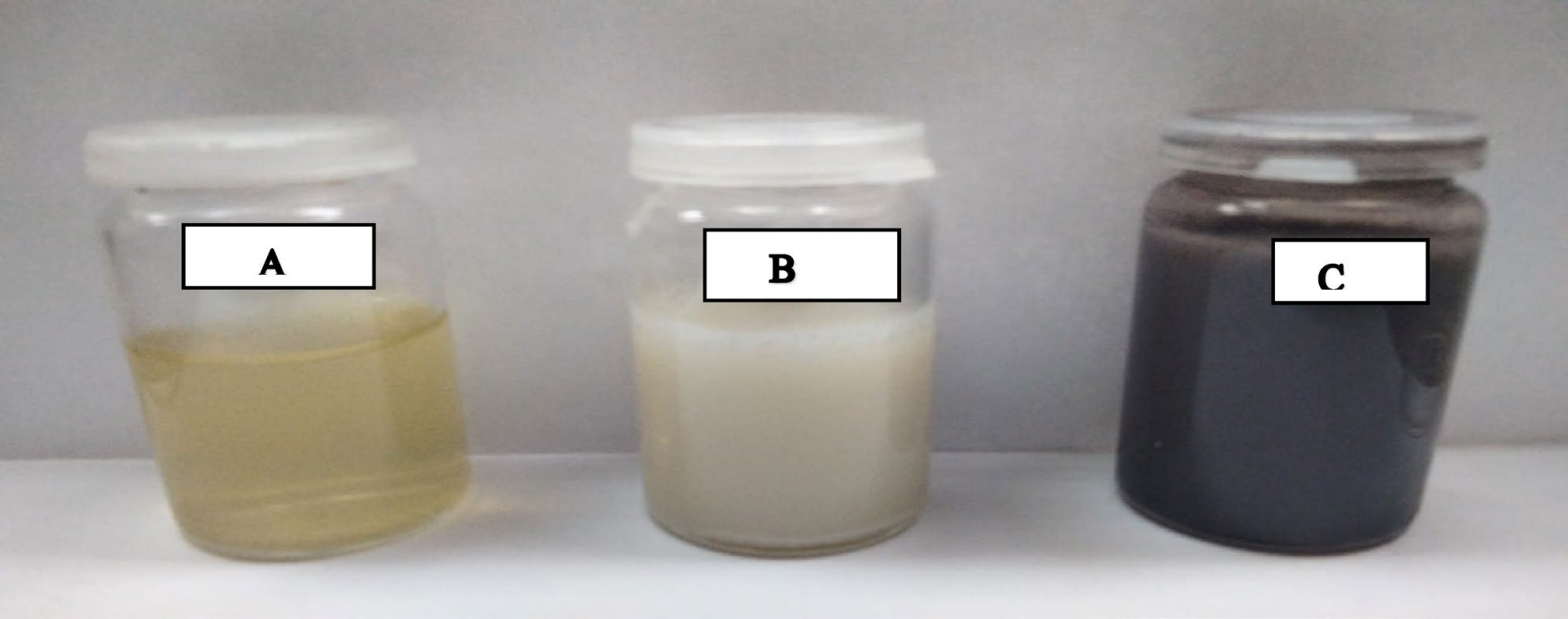
Color change during the bio-reduction of AgNO3 into AgNPs using B*acillus cereus* Nem 212 cell- free filtrate (**A**) *Bacillus cereus* cell- free filtrate before synthesis, (**B**) AgNO3 solution after adding *Bacillus cereus* cell- free filtrate, (**C**) Synthesized silver nanoparticles in dark brown colour solutions after 24 h.

Silver nitrate (1 g) was dissolved in 10 ml of water and then dropped wisely into 1 L of extraction of *Bacillus cereus* cell- free filtrate under vigorous stirring (1000 rpm) at room temperature for 24 h. The addition of aqueous bacteria-extract to colorless silver nitrate solution resulted in a change in the color to dark brown after 24 h (Fig. 6).

### Preparation of garlic oil emulsion (GaO)

One gram of Garlic oil was miscible with 10 ml of ethanol after that the previous solution was completed to 1 L using water.

### Synthesis of Garlic oil nanoemulsion (GaO-nanoemulsion)

Garlic oil (1 g) was dispersed into 1 L of water using Tween 20 (8 g, Sigma Aldrich) as dispersing agent to obtain oil-in-water garlic nanoemulsion solution. This emulsification was done under vigorous stirring (10000 rpm) using High Speed Homogenizer (D-500 Pro; Germany) at room temperature for 15 min.

### Characterization

The average particles size diameter of the samples was measured by using a particle size analyzer (Nano-ZS, Malvern Instruments Ltd., UK). For measuring size distribution, the sample was sonicated for 10–15 min. just before the assessment.

The bio-Ag-NPs were examined in a Philips PW 1830 diffractometer by X-ray diffraction (XRD) through the use of radiation emitting from CuKα (λ = 0.154 nm) at 35 mA and 40 kV. The corrections of dL values determination were carried out at ± 0.01 nm.

The JASCO V 530 (4–21, Sennin-cho 2-chome, Hachioji, Tokyo 193–0835, Japan) model spectrophotometer was used to produce UV-visible absorption spectra in the 400–800 nm spectral region with an accuracy of ± 0.2 nm using quartz cells with a 1 cm path length.

### Nematode identification and field experiment

This field experiment was carried out during January 2024- May 2024 at Kafr- Hakim village, Giza Governorate, Egypt. The experimental field was naturally infested with *Meloidogyne incognita*. The roots of eggplants previously planted in the experimental field were collected and adult females were picked out from the infected roots to identify nematode species using the morphological characteristics of their perennial pattern^68^. The experimental area was divided into 2 plots each comprising 8 rows (6 m. length and 50 cm width) and the distance of (50 cm) between plants. The experiment was set up in a completely randomized block design with 11 treatments, and each treatment was replicated 3 times. The treatments, doses and their abbreviations are shown in (Table 1). The treatments were:1-T1: *M. incognita*- infested non- treated soil (Control). T2-a: Silver nitrate at planting. T2- b: Silver nitrate at planting and after one month. T3-a: Endophytic bacteria *Bacillus cereus* Nem 212 cell free culture filtrate at planting, T3-b: Endophytic bacteria *B. cereus* Nem 212 cell free culture filtrate at planting and after one month. T4-a: Garlic essential oil solution at planting. T4-b: Garlic essential oil solution at planting and after one month. T5-a: bio- silver nanoparticles synthesized by *B. cereus* Nem 212 at planting, T5-b: bio-silver nanoparticles synthesized by *B. cereus* Nem 212 at planting and after one month. T6-a: Garlic oil nanoemulsion at planting. T6-b: Garlic oil nanoemulsion at planting and after one month.

The treatments were added at the rate of 10 ml/ plant as a soil drench and were added at each time / hill. The frequencies of application of the tested bio agents in each bio agent- allocated area were: 1-At planting time, 2- At planting time and after one month. Initial population densities of *M. incognita* juvenile’s j_2_ were determined at planting time^69^ from 250 g. subsamples of well mixed soil from each row, and then all the two plots were treated by the bio control agents according to the allocated area for each treatment. After one month, only plot 2 was treated with the bio control agents. Four months later at harvest five eggplants were randomly selected from each treatment- allocated area, fruits were hand harvested for yield estimation and recorded in terms of their average weights. Other plant growth parameters, such as fresh and dry weights, number of the fruits, number of leaves and flowers were also recorded. For evaluation of nematode reproductive parameters (NRPs), the number of galls and egg masses/ 5 g. roots, as well as number of *M. incognita* juveniles /one g. roots, and total number of eggs/ 5 g. roots were recorded. Assessment of eggs per 5 g. roots: one – gram subsample from 5 g eggplant roots was taken, cut into pieces of 2-cm long. *M. incognita* eggs were extracted from the roots using 0.5% NaOCL solution for 3 min and then obtained by rinsing the egg suspensions with sterile water in a sieve with 25-µm openings according to the method^70^. Eggs were counted under a light microscope and their average numbers were recorded. The final nematode soil population was extracted and densities of *M. incognita* were determined and expressed as the number of juveniles /250 g soil^68^. Percentage nematode reduction was determined according to Henderson and Tilton formula^71^.

Nematode reduction % = {1-(PTA/PTB x PCB/PCA)} x 100

Where PTA = population in treated plot after application, PTB = population in treated plot before application, PCB is population in check plot before application, and PCA = population in check plot after application.

### Statistical analysis

All obtained data were subjected to proper statistical of variance^72^ using Assistant program version 7.6 beta. The means values were compared using Duncan^73^Multiple Range Test at *P* ≤ 0.05 level.

## Electronic supplementary material

Below is the link to the electronic supplementary material.

Supplementary Material 1

Supplementary Material 2

## Data Availability

The datasets generated during the current study are available from the corresponding author on reasonable request. The bacterial isolate was deposited in the GenBank data base as *Bacillus cereus* Nem 212 under accession number OK284601.

## References

[1] FAO stat data. Annual statistical agriculture data for Arab Republic of Egypt C.F. statistical agriculture data of agriculture and reform lands ministry Egypt. (2012).

[2] Abd-Elgawad, M. Biological control of nematodes infecting eggplant in Egypt. *Bull. Natl. Res. Centre* **45** (6). 10.1186/s42269-020-00463-0 (2021).

[3] Hasan, M. & Bai, H. Profitability of Brinjal production in three districts of Bangladesh. *Eco-friendly Agric. J.* **9**, 55–59 (2016).

[4] El-Sayed, G. et al. Overproduction of neutral protease in *Bacillus* 168 through site-directed mutation for biocontrol of *Meloidogyne incognita*. *SABRAO J. Breed. Genet.* **56** (4), 1669–1681. 10.54910/sabrao2024.56.4.32 (2024).

[5] Tariq, M., Ameen, F., Khan, A., Alkahtani, M. D. F. & Siddiqui, M. A. Repellent and nematostatic behaviour of botanical extracts against Root-Knot nematode Meloidogyne incognita attacking Solanum melongena L. *Pol. J. Environ. Stud.* **31** (1), 307–314. 10.15244/pjoes/138178 (2022).

[6] Subedi, S., Thapa, B. & Shrestha, J. Root-knot nematode (*Meloidogyne incognita*) and its management: a review. *J. Agric. Nat. Resour.* **3** (2), 21–31. 10.3126/janr.v3i2.32298 (2020).

[7] Abdelkhalek, A., Salem, M., Hafez, E., Behiry, S. I. & Qari, S. H. The phytochemical, antifungal, and first report of the arrival properties of Egyptian *Haplophylum tuberculatum* extract. *Biology* **9** (9), 248. 10.3390/biology9090248 (2020c). 32854351 10.3390/biology9090248 PMC7565794

[8] Osman, H. et al. Bio-fertilizer′ protocol for controlling root knot nematode *Meloidogyne Javanica* infecting peanut fields. *Egypt. J. Biol. Pest Control*. **31**, 130. 10.1186/s41938-021-00471-w (2021).

[9] Osman, H. et al. Antagonistic potential of an Egyptian entomopathogenic nematode, compost and two native endophytic bacteria isolates against the root knot nematode (*Meloidogyne incognita*) infecting potato under field conditions. *Egypt. J. Biol. Pest Control*. **32**, 137. 10.1186/s41938-022-00635-2 (2022).

[10] D., Addabbo, T., Ladurner, E. & Troccoli, A. Nematicidal activity of a Garlic extract formulation against the grapevine nematode *Xiphinema index*. *Plants* **12** (4), 739. 10.3390/plants12040739 (2023). 36840087 10.3390/plants12040739 PMC9966491

[11] Nazzal, M. & Yass, S. Laboratory evaluation of efficiency of some normal and nano amino acids in hatching eggs and vitality of J_2_ second instar juveniles in root – knot nematode *Meloidogyne* spp. 4th International Conference Modern Technologies in Agricultural Sciences IOP Conference Series: *Earth Environ. Sci.* **1262** (3), 032018. 10.1088/1755-1315/1262/3/032018 (2023).

[12] Nour El-Deen, A. & El–Deeb, B. Effectiveness of silver nanoparticles against root–knot nematode, *Meloidogyne incognita* infecting tomato under greenhouse conditions. *Journal*.

[13] Ghareeb, R. et al. Nematicidal activity of seaweed- synthesized silver nanoparticles and extracts against *Meloidogyne incognita* on tomato plants. *Sci. Rep.* **12** (1), 3841. 10.1038/s41598-022-06600-1 (2022). 35264583 10.1038/s41598-022-06600-1 PMC8907182

[14] Cromwell, W., Yang, J., Starr, J. & Jo, K. Nematicidal effects of silver nanoparticles on root-knot nematode in Bermudagrass. *J. Nematology*. **46** (3), 261–266 (2014). PMCID: PMC4176408 PMID: 25275999. PMC4176408 25275999

[15] Taha, E. & Abo-Shady, N. Effect of silver nanoparticles on the mortality pathogenicity and reproductivity of entomopathogenic nematodes. *Int. J. Zoological Res.* **12**, 47–50. 10.3923/ijzr.2016.47.50 (2016).

[16] Nazir, K., Mukhtar, T. & Javed, H. In vitro effectiveness of silver nanoparticles against root- knot nematode (*Meloidogyne incognita*). *Pakistan J. Zool.* **51** (6), 2077–2083. 10.17582/journal.pjz/2019.51.6.2077.2083 (2019).

[17] Tariq, M., Mohammad, K. N., Ahmed, B., Siddiqui, M. A. & Lee, J. Biological synthesis of silver nanoparticles and prospects in plant disease management. *Molecules* **27** (15), 4754. 10.3390/molecules27154754 (2022). 35897928 10.3390/molecules27154754 PMC9330430

[18] Mohamed, Y., Osman, O., Elshahawy, I., Soliman, G. & Mohamed, A. Charcoal rot and root- knot nematode control on faba bean by photosynthesized colloidal silver nanoparticles using bioactive compounds from *Moringa oleifera* leaf extract. *J. Plant Protect. Res.* **61** (4), 414–429. 10.24425/jppr (2021).

[19] Mohammad, A. et al. Nematicidal activity of sweet annie and garden Cress nano-formulations and their impact on the vegetative growth and fruit quality of tomato plants. *Sci. Rep.* **12**, 22302. 10.1038/s41598-022-26819-2 (2022). 36566273 10.1038/s41598-022-26819-2 PMC9789970

[20] Nassar, A. Effectiveness of silver nanoparticles of extracts of Urtica urens (Urticaceae) against root-knot nematode Meloidogyne incognita. *Asian J. Nematology*. **5** (1), 14–199. (2016).

[21] Hamed, S., Mostafa, A., Abdel- Raouf, N. & Ibraheem, I. Biosynthesis of silver and silver chloride nanoparticles by *Parachiorella Kessien* SAG 211 – 11 and evaluation of its nematicidal potential against the root knot nematode *Meloidogyne incognita*. *Australian J. Basic. Appl. Sci.* **10** (18), 354–364 (2016).

[22] El-Habashy, D. Effectiveness of nanoparticles of some plant extracts against root-knot nematode, *Meloidogyne incognita* on tomato plants. *SVU-International J. Agricultural Sci.* **4** (3), 46–57. 10.21608/svuijas.2022.143524.1213 (2022).

[23] Hamed, S., Hagag, E. & Abd El-Raouf, N. Green production of silver nanoparticles, evaluation of their nematicidal activity against *Meloidogyne Javanica* and their impact on growth of faba bean. *Beni-suef Univ. J. Basic Appl. Sci.* **8** (9). 10.1186/s43088-019-0010-3 (2019).

[24] Pallavi, Mehta, P., Srivastava, R., Arota, S. & Sharma, A. Impact assessment of silver nanoparticles on plant growth and soil bacterial diversity. *3 Biotech* 6254 (2016). 10.1007/s13205-016-0567-7 PMC5125160 28330326

[25] Iqbal, M. et al. Effect of silver nanoparticles on growth of wheat under heat stress. *Iran. J. Sci. Technol. Trans. A Sci.* **43** 387–395. 10.1007/s40995-017-0417-4 (2019).

[26] Pandey, P., Kass, P., Soupir, M., Biswas, S. & Singh, V. Contamination of water resources by pathogenic bacteria. *AMB Express*. **4** (1), 51. 10.1186/s13568-014-0051-x (2014). 25006540 10.1186/s13568-014-0051-x PMC4077002

[27] Hojjat, S. The effect of silver nanoparticles on lentil seed germination under drought stresses. *Int. J. Farm. Allied Sci.* **5** (3), 208–212. (2016).

[28] Dietz, K. & Herth, S. Plant nanotoxicology. *Trends Plant Sci.* **16** (11), 582–589. 10.1016/j.tplants.2011.08.003 (2011). 10.1016/j.tplants.2011.08.003 21906987

[29] A. I. H. A., Hatata, M. & Huqail, A. I. &Ibrahim MM. Preparation, characterization of silver phyto nanoparticles and their impact on growth potential of *lupines termis*. L. seedlings. *Saudi Journal Biol. Sciences*. **25** (2), 313–319. 10.1016/j.sjbs.2017.08.013 (2017). 10.1016/j.sjbs.2017.08.013 PMC5815996 29472784

[30] Riaz, M. et al. Exceptional antibacterial and cytotoxic potency of monodisperse greener AgNPs prepared under optimized pH and temperature. *Sci. Rep.* **11**, 2866. 10.1038/s41598-021-82555-z (2021). 33536517 10.1038/s41598-021-82555-z PMC7858571

[31] Arshad, H., Sami, M., Sadaf, S. & Hassan, U. *Salvadora persica* mediated synthesis of silver nanoparticles and their antimicrobial efficacy. *Sci. Rep.* **11** 5996. 10.1038/s41598-021-85584-w (2021). 10.1038/s41598-021-85584-w PMC7966387 33727607

[32] Kushram, P. & Susmita, B. Improving biological performance of 3D-printed scaffolds with garlic-extract nanoemulsions. *ACS Appl. Mater. Interfaces*. **16** (37), 48955–48968. 10.1021/acsami.4c05588 (2024). 39196793 10.1021/acsami.4c05588

[33] Ardakani, A. Toxicity of silver, titanium and silicon nanoparticles on the root-knot 184 nematode, *Meloidogyne incognita*, and growth parameters of tomato. *Nematology* **15** (6), 671–677 (2013).

[34] Korkmaz, N. & Karadag, A. Microwave assisted green synthesis of ag, Ag2O and Ag2O3 nanoparticles. *J. Turkish Chem. Soc. Sect. A: Chem.* **8** (2), 585–592. 10.18596/jotcsa.784065 (2021).

[35] Heflish, A. et al. Green biosynthesized silver nanoparticles using *Acalypha wilkesiana* extract control root- knot nematode. *J. King Saudi Univ. Sci.* **33** 101516. (2021).

[36] Kumar, A. et al. Potential applications of engineered nanoparticles in plant disease management: A. Critical update. *Chemosphere* **295** 133798. (2022). 10.1016/j.chemosphere.2022.133798 35122813

[37] Hassan, M., Zawam, H., El-Nahas, S. & Desoukey, A. Comparison study between silver nanoparticles and two nematicides against *Meloidogyne incognita* on tomato seedlings. *Plant. Pathol. J.* **15** (4), 144–151. 10.3923/ppj.2016.144.151 (2016).

[38] Jung, W. et al. Antibacterial activity and mechanism of action of the silver ion in *Staphylococcus aureus* and *Escherichia coli*. *Appl. Environ. Microbiol.* **74** (7), 2171–2178. 10.1128/AEM.02001-07 (2008). 18245232 10.1128/AEM.02001-07 PMC2292600

[39] El-Nagdi, W. & Abd-El-Khair, H. Application of *Bacillus* species for controlling root knot nematode *Meloidogyne incognita* in eggplant. *Bull. Natl. Res. Centre*. **43**, 154. 10.1186/s42269-019-0187-6 (2019).

[40] Abbassy, M., Abdel-Rasoul, M., Nassar, A. & Soliman, B. Nematicidal activity of silver nanoparticles of botanical products against root-knot nematode, *Meloidogyne incognita*. *Archives Phytopathol. Plant. Prot.* **50**, 16–17. 10.1080/03235408.2017.1405608 (2017).

[41] Bashan, Y. & de Bashan, L. Bacteria/plant growth promotion. In: (ed Hillel, D.) Encyclopedia of Soils in the Environment. Elsevier, Oxford, 103–115) (2005).

[42] Abd-El-Khair, H., El-Nagdi, W., Youssef, M., Abd-Elgawad, M. & Dawood, M. Protective effect of *Bacillus subtilis B. pumilus* and *Pseudomonas fluorescens* isolates against root knot nematode *Meloidogyne incognita* on Cowpea. *Bull. Natl. Res. Centre* **43** (1). 10.1186/s42269-019-0108-8 (2019).

[43] Osman, H., Ameen, H., Mohamed, M. & Elkelany, U. Efficacy of integrated microorganisms in controlling root-knot nematode *Meloidogyne Javanica* infecting peanut plants under field conditions. *Bull. Natl. Res. Centre*. **44**, 134. 10.1186/s42269-020-00366-0 (2020).

[44] Saharan, B. & Nehra, V. Plant growth promoting rhizobacteria: a critical review. *Life Sci. Med. Res.* **21**, 1–33 (2011).

[45] Xio, T. et al. *Bacillus cereus* X5 enhanced bio-organic fertilizers effectively control root-knot nematodes (*Meloidogyne* sp). *Pedosphere* **23** (2), 160–168. 10.1016/S1002-0160(13)60003-X (2013).

[46] Tian, B., Yang, J. & Zhang, K. Bacteria used in the biological control of plant parasitic nematodes: populations, mechanisms of action, and future prospects. *FEMS Microb. Ecol.* **61:2**, 197–213. 10.1111/j.1574-6941.2007.00349.x (2007). 10.1111/j.1574-6941.2007.00349.x 17651135

[47] Huang, W., Yang, Y., Hu, H., Cao, K. & Zhang, S. Sustained diurnal stimulation of Cyclic electron flow in two tropical tree species *Erythrophleum guineense* and *Khaya ivorensis*. *Frontier Plant. Sci.* **7**, 1068. 10.3389/fpls.2016.01068 (2016). 10.3389/fpls.2016.01068 PMC4950474 27486473

[48] Wiesman, Z. & Chapagain, B. Larvicidal activity of saponin containing extracts and fractions of fruit mesocarp of *Balanites aegyptiaca*. *Fitoterapia* **77**, 420–424 (2006). 16814957 10.1016/j.fitote.2006.05.012

[49] Soliman, G. et al. Anti- nemic potential of *Laurenica papillosa* and *Dilophys fasciola* -biosynthesized nano-extracts against tomato root knot nematode. *Meloidogyne Incognita Phytoparasitica*. **52**, 37. htpps:// doi.org/10. 1007/s 12600-024-01157-3 (2024).

[50] Lim, D., Roh, J., Eom, H., Hyun, J. & Choi, J. Oxidative stress- related PMK- I P38 MAPK activation as a mechanism for toxicity of silver nanoparticles to reproduction in the nematode *Caenorhabditis elegans*. *Environ. Toxicol. Chem.* **31** (3), 585–592 (2012). 22128035 10.1002/etc.1706

[51] Sharon, M., choudhary, A. K. & Kumar, R. Nanotechnology in agricultural diseases and food safety. *J. Phytology*. **2**, 83–92 (2010).

[52] Roh, J. et al. Ecotoxicity of silver nanoparticles on the soil nematode Caenorhabditis elegans using functional ecotoxicogenomics. *Environ. Sci. Technol.* **43**, 3933–3940 (2009). 19544910 10.1021/es803477u

[53] Yasmeen, B., Islam, S., Uddin, M. & Jahan, R. Prevalence of urinary tract infection, its causative agents and antibiotic sensitivity pattern: A study in northern International Medical College Hospital, Dhaka. *North. Int. Med. Coll. J.* **7** (1), (2015).

[54] Craigie, J. Seaweed extracts stimuli in plant science and agriculture. *J. Appl. Phycol.* **23** (2008), 371–393. 10.1007/s10811-10-9560-4 (2011).

[55] Jardim, I., Oliveira, D. & Campos, V. Garlic essential oil reduces the population of *Meloidogyne incognita* in tomato plants. *Eur. J. Plant Pathol.* **157** (2), 1–13. 10.1007/s10658-020-02000-1 (2020).

[56] El-Saedy, M., Mokbel, A. & Hammad, S. Efficacy of plant oils and Garlic cultivation on controlling *Meloidogyne incognita* infected tomato plants. *Pakistan J. Nematololgy*. **32** (1), 39–50 (2014).

[57] Huang, Q., Yu, H. & Ru, Q. Bioavailability and delivery of nutraceuticals using nanotecnology. *J. Food Sci.* **75** (1), 50–57. 10.1111/j.1750-3841.2009.01457.x (2010). 10.1111/j.1750-3841.2009.01457.x 20492195

[58] Hammad, E. & Hasanin, M. Antagonistic effect of nanoemulsions of some essential oils against *Fusarium exysporum* and root – knot nematode *Meloidogyne javanica* on coleus plants. Pakistan *J. Nematol.* **40** (1), 35–48. 10.17582/journal.pin/2022/40.1.35.48 (2022).

[59] Laquale, S., Candido, V., Avato, P., Aregentieri, M. & Addabbo, T. Essential oils as soil biofumigants for the control of the root- knot nematode *Meloidogyne incognita* on tomato. *Annals Appl. Biology*. **167** (2), 217–224. 10.1111/aab.12221 (2015).

[60] Mendes, J. et al. Chemical composition and antibacterial activity of Eugenia brejoensis essential oil nano emulsions against Pseudomonas fluorescens. *LWT* **93** (3), 659–564. 10.1016/j.lwt.2018.04.015 (2018).

[61] Kokalis-burelle, N., Rodriguez-Kabana, R. & Klop-Per, J. Organic amendments and natural chemicals as components of transplant mixes for control of root knot nematode. *Phytopathology* **89** (6 Sup), S41 (1999).

[62] Sarkar, S. Incidental finding of root knot symptoms in *Iavandula angustifolia* mill: first report from India. *J. Med. Plants Stud.* **8** (4), 292–299 (2020).

[63] Anton, N. & Vandamme, T. Nanoemulsions and micro-emulsions: clarifications of the critical differences. *Pharm. Res.* **28**, 978–985. https://doi.org/10.1007/s 11095-010-0309-1 (2011). 21057856 10.1007/s11095-010-0309-1

[731] Barbosa, P. et al. Nematicidal activity of essential oils and volatiles derived from Portuguese aromatic flora against the Pinewood nematode, *Bursaphelenchus Xylophilus*. *J. Nematology*. **42** (1), 8–16 (2010). PMC3380513 22736831

[65] Sharon, M., Choudhary, A. & Kumar, R. Nanotechnology in agricultural diseases and food safety. *J. Phytology*. **2** (4), 83–92 (2010). ISSN: 2075-6240.

[66] Dubre, N., Petica, A., Buda, M., Anical, I. & Visan, T. Electrochemical synthesis of silver nanoparticles in Aquas electrolytes. *UPB Sci. Bull.* **76** (4), 127–136 (2014).

[67] Davis, R., Botstein, D. & Rothho, J. Transfection of (DNA) in bacterial genetics: a manual for genetic engineering advanced bacterial genetic. *Cold Spring Harbor New. York*. **67**, 134–137 (1980).

[68] Taylor, A. & Sasser, J. *Biology, Identification and Control of Root Nematodes (Meloidogyne species).* (IMP. North Carolina State University Graphics, 1978).

[69] Barker, T. Nematode extraction and bioassays Pp. in: (eds Barker, Y. R., Carter, C. C. & Sasser, J. N.) An Advanced Treatise on *Meloidogyne*, 11. North Carolina State University, Raleigh, 19–35 (1985).

[70] Hussey, R. & Barker, K. A comparison of methods of collecting inoculums of *Meloidogyne* spp. *Plant. Disease Report.* **57**, 1025–1028 (1973).

[71] Puntener, W. Manual for field trials in plant protection. Basel, Switezerland agricultural division. *Ciba Geigy Limited* **205** (1981).

[72] Snedecor, G. & Cochran, W. Statistical methods, 7th edn. *Lowa state university Press* 507 (1980).

[73] Duncan DB A significant test for differences between ranked treatments in analysis of variance. *Va. J. Sci.* **2**, 171–189 (1955).

