# Supplementary material for: Comparative field study of silver nanoparticles and garlic oil nanoemulsion for nematode control and yield enhancement in eggplant
Source: Sci Rep. 2025 Jun 20;15:20220. doi: 10.1038/s41598-025-06697-0 (PMC12181296; doi:10.1038/s41598-025-06697-0)
Supplement: Supplementary file 1 — Supplementary Material 1 [file 41598_2025_6697_MOESM1_ESM.docx]

Table 1: Treatments designed for controlling root-knot nematode *Meloidogyne incognita* infecting eggplant CV. Baladi under field conditions.

| Treatments  number | Frequency  of  application | Treatment full name | Application rate | | Treatments Abbreviation |
| --- | --- | --- | --- | --- | --- |
|  |  | A-Normal formulations |  |  |  |
| T1 control |  | Untreated infected control (negative control |  |  |  |
| T2- a  b | 1  2 | Silver nitrate solution at planting  Silver nitrate solution at planting and after one month | 10ml/plant  10ml/plant | | AgNO_3_ |
| T3- a  b | 1  2 | Endophytic bacteria *Bacillus cereus* Nem 212 Cell free culture filtrate at planting  Endophytic bacteria *Bacillus cereus* Nem 212 Cell free culture filtrate at planting and after one month | 10ml/plant  10ml/plant | | *B.cereus* cell free filtrate |
| T4- a  b | 1  2 | Garlic essential oil emulsion at planting  Garlic essential oil emulsion at planting and after one month | 10ml/plant  10ml/plant | | garlic oil solution |
| B- Nanoparticles formulations (Dose, and time of application as normal formulation) | | | | | |
| T 5- a  b | 1  2 | Bio-silver nanoparticles synthesized by Bacillus cereus Nem 212 filtrate at planting  Bio-silver nanoparticles synthesized by Bacillus cereus Nem 212 filtrate at planting and after one month | | 10ml/plant  10ml/plant | bio-silver nanoparticles  Bio - Ag-NPs |
| T6- a    b | 1  2 | Garlic oil nanoemulsion at planting  Garlic oil nanoemulsion at planting and after one month | | 10 ml/plant  10 ml/plant | garlic oil nanoemulsion  GaO- nanoemultion |

Frequency of application: 1- at planting time 2- at planting time and after one month

Table 2: Effects of endophytic bacteria extracts in both normal and bio-silver nanoparticles, garlic oil solution and nanoemulsion on eggplant CV. Baladi infested with *Meloidogyne incognita* under field conditions.

| Treatments | *Freq. of  app. | Initial pop/  250 g soil | Final pop./**%  250g soil Red | | No. of % Red.  Juveniles  / 5g root | | No. of % Red.  galls/  5g roots | | No of egg %Red.  masses/ 5g  roots | | Total eggs/ %Red  5g roots | |
| --- | --- | --- | --- | --- | --- | --- | --- | --- | --- | --- | --- | --- |
| T1 |  | 747a | 2493 a | -- | 302 a | -- | 484 a | -- | 369 a | --- | 212913 a | -- |
| T2-a | 1 | 760 a | 1613 c | 26.3 | 167bc | 44.7 | 136 de | 71.9 | 128 cd | 65.3 | 60672bc | 71.5 |
| b | 2 | 726 a | 1339 d | 46.3 | 163bc | 46.0 | 133 de | 72.5 | 113 cd | 69.3 | 54240bc | 74.0 |
| T3-a | 1 | 750 a | 1927 b | 22.7 | 205 b | 32.1 | 419 b | 15.5 | 314 a | 14.9 | 164850 b | 22.5 |
| b | 2 | 758 a | 1664 c | 33.2 | 178bc | 41.0 | 316 c | 34.7 | 227 b | 38.4 | 111684bc | 47.5 |
| T4-a | 1 | 750 a | 1667 c | 33.1 | 169bc | 44.0 | 179 d | 63.0 | 160 c | 56.6 | 48560 bc | 63.1 |
| b | 2 | 747 a | 1267de | 41.2 | 125 cd | 58.6 | 154 de | 68.2 | 145 cd | 60.7 | 66120 cd | 68.9 |
| T5-a | 1 | 753 a | 1351 d | 45.8 | 157bc | 48.0 | 134 de | 72.3 | 122 cd | 66.9 | 58316 cd | 72.6 |
| b | 2 | 758 a | 1085 e | 56.4 | 125 cd | 58.6 | 111ef | 77.1 | 102 cd | 72.3 | 45696 cd | 78.5 |
| T6-a | 1 | 761 a | 1431 cd | 42.5 | 132 cd | 56.3 | 161 de | 66.7 | 128 cd | 65.3 | 40320bc | 81.0 |
| b | 2 | 762 a | 1068 e | 57.2 | 96 d | 68.2 | 96 f | 80.2 | 73 d | 80.2 | 31536 d | 85.1 |

Each value represents mean of five replicates. Mean followed by the same letter (s) within a column are not significantly (P ≥ 0.05) different according Duncan’s multiple range test. *Freq. of app.: Frequency of application: 1- at planting 2- at planting and afterone month. ** % Red: % reduction.

1-T1: Control. T2-a: Silver nitrate at planting. T2- b: Silver nitrate at planting and after one month. T3-a : Endophytic bacteria *Bacillus cereus* Nem 212 cell free culture filtrate at planting, T3-b: Endophytic bacteria *B. cereus* Nem 212 cell free culture filtrate at planting and after one month. T4-a: Garlic oil emulsion at planting. T4-b: Garlic oil emulsion at planting and after one month. T5-a: bio- silver nanoparticles synthesized by *B.cereus* Nem 212 at planting, T5-b: bio-silver nanoparticles synthesized by *B. cereus* Nem 212 at planting and after one month. T6-a: Garlic oil nanoemulsion at planting. T6-b: Garlic oil nanoemulsion at planting and after one month.

Fig1 : Effects of endophytic bacteria extracts in both normal and bio-silver nanoparticles, garlic oil solution and nanoemulsion on eggplant CV. Baladi infested with *Meloidogyne incognita* under field conditions.

Table3: Effects of endophytic bacteria extracts in both normal and bio -silver nanoparticles, garlic oil emulsion and nanoemulsion on eggplant growth parameters CV. Baladi infested with *Meloidogyne incognita* under field conditions.

| *Tre. | **Freq  Of  app. | Plant shoot  height  (cm) | ***%  Inc. | Root  height  (cm) | ***%  Inc. | Plant  fresh  weight  (g) | ***%  Inc. | Plant ***%  dry Inc  weight  (g) | | No. of *** %  Leaves Inc.  /plant | | No. of *** %  Fruits/ Inc.  Plant | | Fruits **** %  weight/ change.  plant (g) | | No. of ***%  Flowers Inc  / plant | | Yield/ ton/  Feddan | *** %  Inc |
| --- | --- | --- | --- | --- | --- | --- | --- | --- | --- | --- | --- | --- | --- | --- | --- | --- | --- | --- | --- |
| T1 |  | 19 d | -- | 12 e | -- | 48 e | -- | 12 b | -- | 13 a | -- | 8 d | -- | 750 cd | -- | 7 e | -- | 16cd | - |
| T2- a  b | 1  2 | 23 cd  25 cd | 21.0  31.5 | 16 cd  14e | 33.3  16.0 | 72 de  89 cd | 50.0  85.4 | 12 b  16 b | --  33.3 | 19 cd  21cd | 46.1  16.5 | 16 cd  18 cd | 100.0  125.0 | 547e  650 de | -27.0  -13.3 | 10 cd  12 cd | 42.8  71.4 | 11.0 e  13.0 de | -31.3  -18.8 |
| T3- a  b | 1  2 | 21 cd  24 cd | 10.5  26.3 | 16 de  18 cd | 33.3  50.0 | 72 de  95 cd | 50.0  47.9 | 12 b  17 b | 33.3  41.6 | 17 ae  22 cd | 23.5  69.2 | 16 cd  29 bc | 100.0  175.0 | 813 cd  900 c | 8.4  20.0 | 9.0 de  13 bc | 28.5  85.7 | 16.5cd  19.5 c | 3.1  22.0 |
| T4-a  b | 1  2 | 32 bc  38 b | 68.4  100 | 22 ab  22 ab | 83.3  83.3 | 154 b  154 b | 220.8  220.8 | 17 b  23 b | 41.6  91.6 | 26 bc  28 bc | 100.0  115.3 | 29 bc  47 a | 262.5  487.5 | 1216 b  1247 b | 62.1  66.3 | 17 b  21 a | 142.8  200.0 | 25.0 b  26.3 b | 56.3  64.4 |
| T5- a  b | 1  2 | 30 bc  33 bc | 57.9  73.7 | 19 bc  16 cd | 58.3  33.3 | 91 cd  113 c | 89.5  125.4 | 16 b  16 b | 33.0  33.3 | 25 bc  29 bc | 92.1  123.0 | 21 bc  39 bc | 162.5  387.0 | 910 c  950 c | 21.3  27.0 | 14 bc  17 b | 100.0  142.8 | 20.0 cd  24.3 de | 25.0  51.8 |
| T6- a  b | 1  2 | 38 b  54 a | 100.0  184.2 | 25 a  24 ab | 108.3  100.0 | 167 ab  181 a | 247.9  277.0 | 17 b  99 a | 41.6  141.6 | 32 ab  39 a | 146.2  200.0 | 46 a  56 a | 475.0  600.0 | 1400ab  1757 a | 86.6  107.6 | 21 a  25 a | 200.0  257.0 | 28.0 ab  31.5 a | 75.0  96.8 |

Each value represents mean of five replicates. Mean followed by the same letter(s) within a column are not significantly (p ≥ o. o5) different according Duncan' s multiple range test *Tre.: Treatments ** Freq. of app. Frequency of applications:1- at planting 2- at planting and after one month. *** % Inc= % increase ****% change: % change of control1-T1: Control. T2-a: Silver nitrate at planting. T2- b: Silver nitrate at planting and after one month. T3-a: Endophytic bacteria *Bacillus cereus* Nem 212 cell free culture filtrate at planting, T3-b: Endophytic bacteria *B. cereus* Nem 212 cell free culture filtrate at planting and after one month. T4-a: Garlic oil emulsion at planting. T4-b: Garlic oil emulsion at planting and after one month. T5-a: bio- silver nanoparticles synthesized by *B.cereus* Nem 212 at planting, T5-b: bio-silver nanoparticles synthesized by *B. cereus* Nem 212 at planting and after one month, T6-a: Garlic oil nanoemulsion at planting. T6-b: Garlic oil nanoemulsion at planting and after one month.

Fig 2: Effects of endophytic bacteria extracts in both normal and bio -silver nanoparticles, garlic oil emulsion and nanoemulsion on eggplant growth parameters CV. Baladi infested with *Meloidogyne incognita* under field conditions.
