# Supplementary material for: Comparative field study of silver nanoparticles and garlic oil nanoemulsion for nematode control and yield enhancement in eggplant
Source: Sci Rep. 2025 Jun 20;15:20220. doi: 10.1038/s41598-025-06697-0 (PMC12181296; doi:10.1038/s41598-025-06697-0)
Supplement: Supplementary file 2 — Supplementary Material 2 [file 41598_2025_6697_MOESM2_ESM.docx]

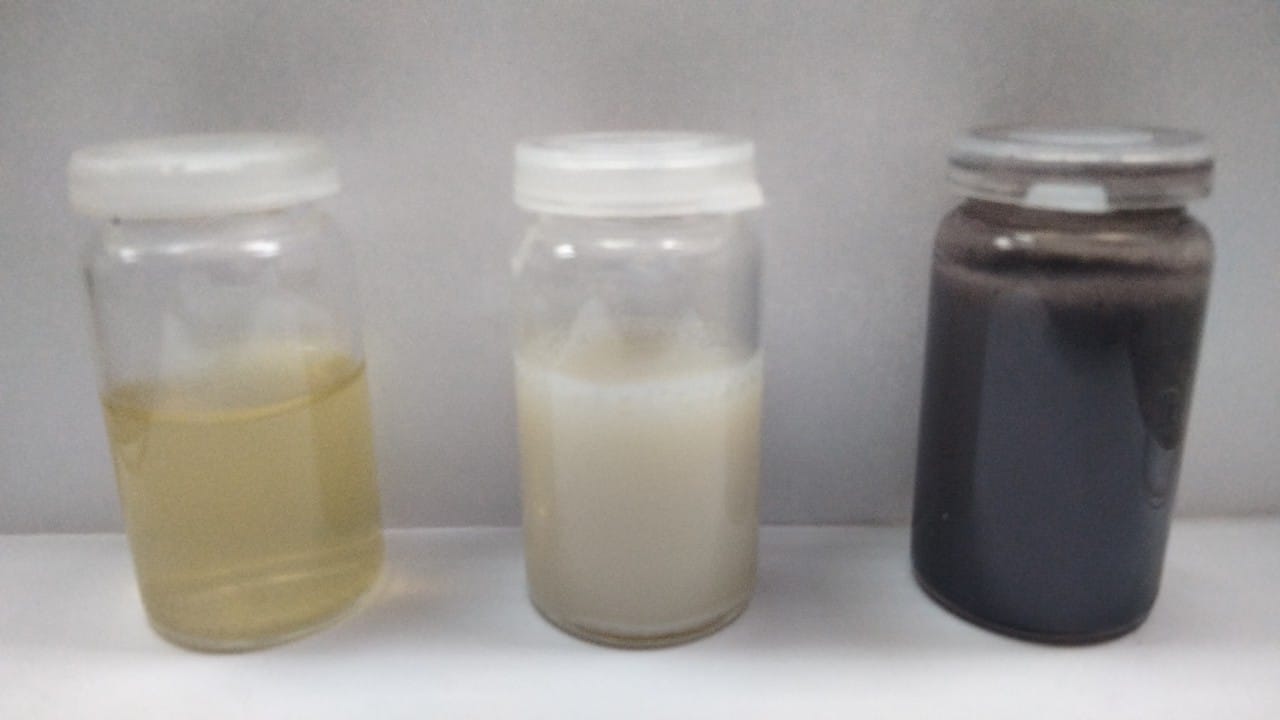


A

Figure 3:Color change during the bio-reduction of AgNO3 into AgNPs using B*acillus cereus* Nem 212 cell- free filtrate (**A**) *Bacillus cereus* cell- free filtrate before synthesis, (**B**) AgNO3 solution after adding *Bacillus cereus* cell- free filtrate, (**C**) Synthesized silver nanoparticles in dark brown colour solutions after 24 hours.

C

B

Figure 4A: Particle size distribution of bio- Ag-NPs

Figure 4B: Particle size distribution of GaOnanoemulsions

Figure 5A: UV-Vis spectrum of bio- Ag-NPs

Figure 5B: UV-Vis spectrum of Garlic oil solution and Garlic oil nanoemulsion

Fig. 6: XRD pattern of bio-Ag-NPs.
